# Longitudinal immune profiles in type 1 leprosy reactions in Bangladesh, Brazil, Ethiopia and Nepal

**DOI:** 10.1186/s12879-015-1128-0

**Published:** 2015-10-28

**Authors:** Saraswoti Khadge, Sayera Banu, Kidist Bobosha, Jolien J. van der Ploeg-van Schip, Isabela M. Goulart, Pratibha Thapa, Chhatra B. Kunwar, Krista E. van Meijgaarden, Susan J.F. van den Eeden, Louis Wilson, Senjuti Kabir, Hymonti Dey, Luiz R. Goulart, Janaina Lobato, Washington Carvalho, Yonas Bekele, Kees L.M.C. Franken, Abraham Aseffa, John S. Spencer, Linda Oskam, Tom H.M. Otttenhoff, Deanna A. Hagge, Annemieke Geluk

**Affiliations:** Dept. of Infectious Diseases, Leiden University Medical Center (LUMC), PO Box 9600, 2300 RC Leiden, The Netherlands; Mycobacterial Research Laboratories, Anandaban Hospital, Kathmandu, Nepal; Armauer Hansen Research Institute, Addis Ababa, Ethiopia; International Center for Diarrhoeal Disease Research Bangladesh, Dhaka, Bangladesh; National Reference Center for Sanitary Dermatology and Leprosy, Faculty of Medicine, Federal University of Uberlandia, Minas Gerais, Brazil; KIT Biomedical Research, Royal Tropical Institute, Amsterdam, The Netherlands; Dept. of Microbiology, Immunology and Pathology, Colorado State University, Fort Collins, USA

**Keywords:** Biomarkers, Cytokines, Diagnostics, Leprosy, M. leprae, Ratios, Reactions

## Abstract

**Background:**

Acute inflammatory reactions are a frequently occurring, tissue destructing phenomenon in infectious- as well as autoimmune diseases, providing clinical challenges for early diagnosis. In leprosy, an infectious disease initiated by *Mycobacterium leprae* (*M. leprae*), these reactions represent the major cause of permanent neuropathy. However, laboratory tests for early diagnosis of reactional episodes which would significantly contribute to prevention of tissue damage are not yet available.

Although classical diagnostics involve a variety of tests, current research utilizes limited approaches for biomarker identification. In this study, we therefore studied leprosy as a model to identify biomarkers specific for inflammatory reactional episodes.

**Methods:**

To identify host biomarker profiles associated with early onset of type 1 leprosy reactions, prospective cohorts including leprosy patients with and without reactions were recruited in Bangladesh, Brazil, Ethiopia and Nepal. The presence of multiple cyto-/chemokines induced by *M. leprae* antigen stimulation of peripheral blood mononuclear cells as well as the levels of antibodies directed against *M. leprae*-specific antigens in sera, were measured longitudinally in patients.

**Results:**

At all sites, longitudinal analyses showed that IFN-γ-, IP-10-, IL-17- and VEGF-production by *M. leprae* (antigen)-stimulated PBMC peaked at diagnosis of type 1 reactions, compared to when reactions were absent. In contrast, IL-10 production decreased during type 1 reaction while increasing after treatment. Thus, ratios of these pro-inflammatory cytokines versus IL-10 provide useful tools for early diagnosing type 1 reactions and evaluating treatment. Of further importance for rapid diagnosis, circulating IP-10 in sera were significantly increased during type 1 reactions. On the other hand, humoral immunity, characterized by *M. leprae-*specific antibody detection, did not identify onset of type 1 reactions, but allowed treatment monitoring instead.

**Conclusions:**

This study identifies immune-profiles as promising host biomarkers for detecting intra-individual changes during acute inflammation in leprosy, also providing an approach for other chronic (infectious) diseases to help early diagnose these episodes and contribute to timely treatment and prevention of tissue damage.

**Electronic supplementary material:**

The online version of this article (doi:10.1186/s12879-015-1128-0) contains supplementary material, which is available to authorized users.

## Background

Leprosy is a chronic, immunoregulatory infectious disease caused by *Mycobacterium leprae* that particularly affects the skin and peripheral nerves and often results in severe, life-long disabilities and deformities [1, 2]. The number of new cases has plateaued at 220,000–250,000 annually, but many linger undetected [3, 4]. Leprosy remains endemic in Africa, South America and Asia and with increasing migration, new cases are detected in developed countries, where initial misdiagnosis is likely to occur [5–7].

The inter-individual variability in clinical manifestations of leprosy closely parallels the ability of the host to mount an effective immune response to *M. leprae*. This is depicted by an immunological and clinical spectrum in those who progress to disease, ranging between two completely different poles i.e. tuberculoid (TT) and lepromatous (LL) leprosy [8]. Host resistance to *M. leprae* is associated with the emergence of a protective Thelper-1 (Th1)-based response characterized by the secretion of the innate and adaptive cytokines IL-12p70, IFN-γ, lymphotoxin-α/β, and (moderate levels of) other pro-inflammatory cytokines such as TNF-α. LL patients secrete predominantly anti-inflammatory mediators such as IL-10, accompanied by the absence of Th1-associated cytokines in response to *M. leprae* but characterized by high anti-*M. leprae* antibody titers. Conversely, TT patients produce exacerbated levels of pro-inflammatory cytokines, including those produced by Th17 rather than Th1, and frequently driven by strong innate immune activation resulting in the release of IL-1β and/or IL-6, TGF-β and IL-23 [9, 10].

Although leprosy can be treated effectively with multidrug therapy (MDT), it is complicated by persisters [11] as well as acute inflammatory episodes called leprosy reactions. These immunological complications, occurring before, during and after MDT treatment in 30–50 % of the patients, represent the major cause of leprosy-related neurological damage [12, 13]. Two types of reactions are recognized: type 1 or reversal reactions (RRs) and type 2 or erythema nodosum leprosum (ENL). RRs are considered a delayed hypersensitivity reaction with characteristic infiltrations of skin and nerve lesions by CD4^+^ T-cells producing IFN-γ and TNF-α [14–16]. Up to 30 % of leprosy patients are affected by RRs, which most commonly occur in borderline forms of leprosy (borderline-tuberculoid (BT), borderline-borderline (BB), borderline-lepromatous (BL)) in which concomitant immunological fluctuations can generate significant neuropathology [17]. Prompt diagnosis and anti-reactional treatment contributes to recovery significantly thus reducing risks for permanent tissue damage [18, 19]. Unfortunately, reactions are frequently misdiagnosed due to decreased expertise within integrated health services [17]. Therefore, reliable tests for early diagnosis of RR could make huge differences in clinical outcomes. A major obstacle to developing such tests is the lack of dependable biomarkers for reactions across endemic populations.

For the complex host immuno-pathogenicity of leprosy [2, 14], assessment of multiple rather than single biomarkers is more informative of the hosts’ immune status. Therefore, we aimed to identify relevant host immune-biomarkers for early diagnosis of type 1 reactions. We recruited newly diagnosed leprosy patients longitudinally and studied *M. leprae*-specific cellular- and humoral immunity in blood of patients 1) in the absence of any clinical signs of reactions at least three months before reactions, 2) very early after clinical presentation of reactions and 3) after completion of treatment. Non-reactional patients (before and after treatment) as well as healthy individuals from the same area were analyzed similarly. To accommodate worldwide applicability, independent of the genetic and environmental background, this study was executed similarly in four distinct, prospective cohorts in Asia, Africa and South-America.

## Materials and methods

### General study-procedure

Recruitment took place in Bangladesh (International Centre for Diarrhoeal Disease Research Bangladesh, Dhaka), Brazil (National Reference Centre for Sanitary Dermatology and Leprosy, Uberlandia), Ethiopia (ALERT hospital and Health Centre,) and Nepal (Mycobacterial Research Laboratories, Kathmandu). Experiments were performed according to standard operating procedures and each site was provided with identical reagents.

### Study participants

Patients and endemic controls (EC) were recruited on a voluntary basis between February 2008-March 2013 (Table 1). Leprosy was diagnosed based on clinical, bacteriological and histological observations and classified by skin biopsies according to Ridley and Jopling [1]. Leprosy patients were treated according to WHO standards. Clinical monitoring for reactions was performed during monthly clinic visits. Clinical and demographic data was collected in clinical research forms (Additional file 1) and subsequently transferred in databases with special emphasis on standardizing data collection and definition of reaction between all cohorts [20, 21]. For patients who presented with reactions the type, severity, skin- and/or nerve involvement, number of lesions and relapse were noted, according to state-of-the-art clinical expertise and international consensus scoring [21, 22]. EC were assessed for the absence of clinical signs and symptoms of leprosy and TB. Staff of leprosy- or TB clinics were excluded.

**Table 1:** Participating study sites and study groups

Site	Category^a^	Mean BI^b^	Sex ratio	Age range	Total^c^
			(M/F)	(yr)	
Bangladesh	EC	na^d^	0.9	20–40	20
	BL/LL	2.20	5	18–61	31
	RR	1.68	2.5	21–63	20
Brazil	EC	na^d^	1.3	24–76	23
	BL/LL	1.51	1	22–26	25
	RR	1.95	3.3	25–68	20
Ethiopia	EC	na^d^	1.8	18–45	11
	BL/LL	1.25	1.7	18–52	25
	RR	0.46	2.8	18–60	15
Nepal	EC	na^d^	3.6	19–28	20
	BL/LL	2.96	2	35–58	13
	RR	1.45	2.5	27–50	20

^a^ *EC* endemic control, *BL/LL* borderline leprosy/ lepromatous leprosy, *TT/BT* tuberculoid leprosy/ borderline tuberculoid leprosy, *RR* reversal reaction (type 1 reaction)

^b^ *BI* bacterial index (mean)

^c^Total number of recruited individuals is indicated; samples for multiple time points were not always included. For multiplex cytokine analysis or UPLC-ESI-TOF MS a selected sample size was used for analysis

^d^not applicable

### Leprosy prevalence

Dhaka, prevalence: 2.45/10,000, new case detection rate (NCDR): 0.31/10,000 (Annual Reports of Leprosy Control Institute & Hospital, Dhaka); Uberlandia, prevalence: 0.96/10,000, NCDR: 1,12/10,000 (National Disease Surveillance System, Secretariat of Health Surveillance, Ministry of Health Brazil); Addis Ababa, prevalence: 0.6/10,000 in 2010–2011, 0.4/10,000 in 2012, NCDR: 0.35/10,000 (FMOH reports); Kathmandu, prevalence: 1.1-0.79/10,000, NCDR: 1.67- 1.15/10,000 (Annual Report 2012–2013, Leprosy Control Division, Department of Health Services, Kathmandu).

### Recruitment

Newly diagnosed, untreated leprosy patients without clinical reactions were enrolled and blood was drawn before MDT (t = 0). Patients who presented reactions within three months of the start of therapy were excluded to avoid profile analyses of patients with latent reactions. If patients presented with reactions after more than three months of MDT, blood was drawn before initiation of anti-reactional therapy (t = x). Newly diagnosed leprosy patients who visited clinics with RR were recruited (t = x) but consequently lacked t = 0 samples. From all patients, blood was collected after MDT and/or steroid therapy (t = end). For patients with RR this was done at least one month after completion of steroid therapy to avoid assessment of the effect of steroids. All patients were assessed for the absence of reactions three months after t = end. For patients showing clinical signs of reactions within three months after t = end, this time point was excluded. In case patients died, moved or withdrew from the study, preventing follow-up, their samples were excluded. Blood was used for isolation of peripheral blood mononuclear cells (PBMC). Supernatants and sera were stored at −20 °C.

### Antigens

*M. leprae* recombinant proteins were produced as described [23]. *M. leprae* whole cell sonicate was provided through the NIH/NIAID “Leprosy Research Support” Contract N01 AI-25469 (http://www.beiresources.org).

### Cytokine/chemokine analysis

PBMC, freshly isolated from venous blood, were cultured for 6 days with antigens as described [23]. IFN-γ was determined by ELISA (U-CyTech, Utrecht, The Netherlands) [24]. A positive, reference supernatant was provided to all laboratories. IL-1β, IL-2, IL-4, IL-5, IL-6, IL-7, IL-8, IL-10, IL-12p70, IL-13, IL-17A, IFN-γ, IP-10, G-CSF, GM-CSF, MCP-1, MIG, MIP-1β and TNF in supernatants or sera were measured using the Bio-Plex-suspension-array-system (Bio-Rad, Veenendaal, NL) [23]. IFN-β was determined in undiluted sera (25ul) using Procartaplex IFN-β simplex-kit (eBioscience, Hatfield, UK) and CCL18 was determined (1:10 dilutions; 100 μl) by ELISA (DY394 DuoSet, R & D Systems, Minneapolis, MN) according to manufacturers’ instructions.

### Serology

Antibodies against ML2028 (Ag85B) and ND-O-BSA, a synthetic analogue of phenolic glycolipid I (PGL-I), were determined as described [25].

### Ethics

This study was performed according to the Helsinki Declaration (2008 revision). Participants were informed about the study-objectives, the samples and their right to refuse to take part/withdraw from the study without consequences for their treatment. Written informed consent was obtained before enrolment. All patients received treatment according to national guidelines. Ethical approval of the study-protocol was obtained through appropriate ethics committees: Ethical Review Committee of ICDDR, B (#PR-10032; #PR-2007-069); Brazilian National Council of Ethics in Research (CONEP) and UFU Research Ethics Committee (#499/2008); National Health Research Ethical Review committee Ethiopia (NERC # RDHE/127-83/08); Nepal Health Research Council (NHR #751).

### Statistical analysis

Differences in cytokine concentrations were analysed with two-tailed Mann–Whitney U tests (unpaired samples) for non-parametric distribution and Wilcoxon matched-pairs signed rank test or paired t test for longitudinal analyses using GraphPad Prism version 5.01 for Windows (GraphPad Software, San Diego, CA, USA; www.graphpad.com). The statistical significance level used was *p* < 0.05.

## Results

### Recruitment of four prospective cohorts

**Fig. 1:**
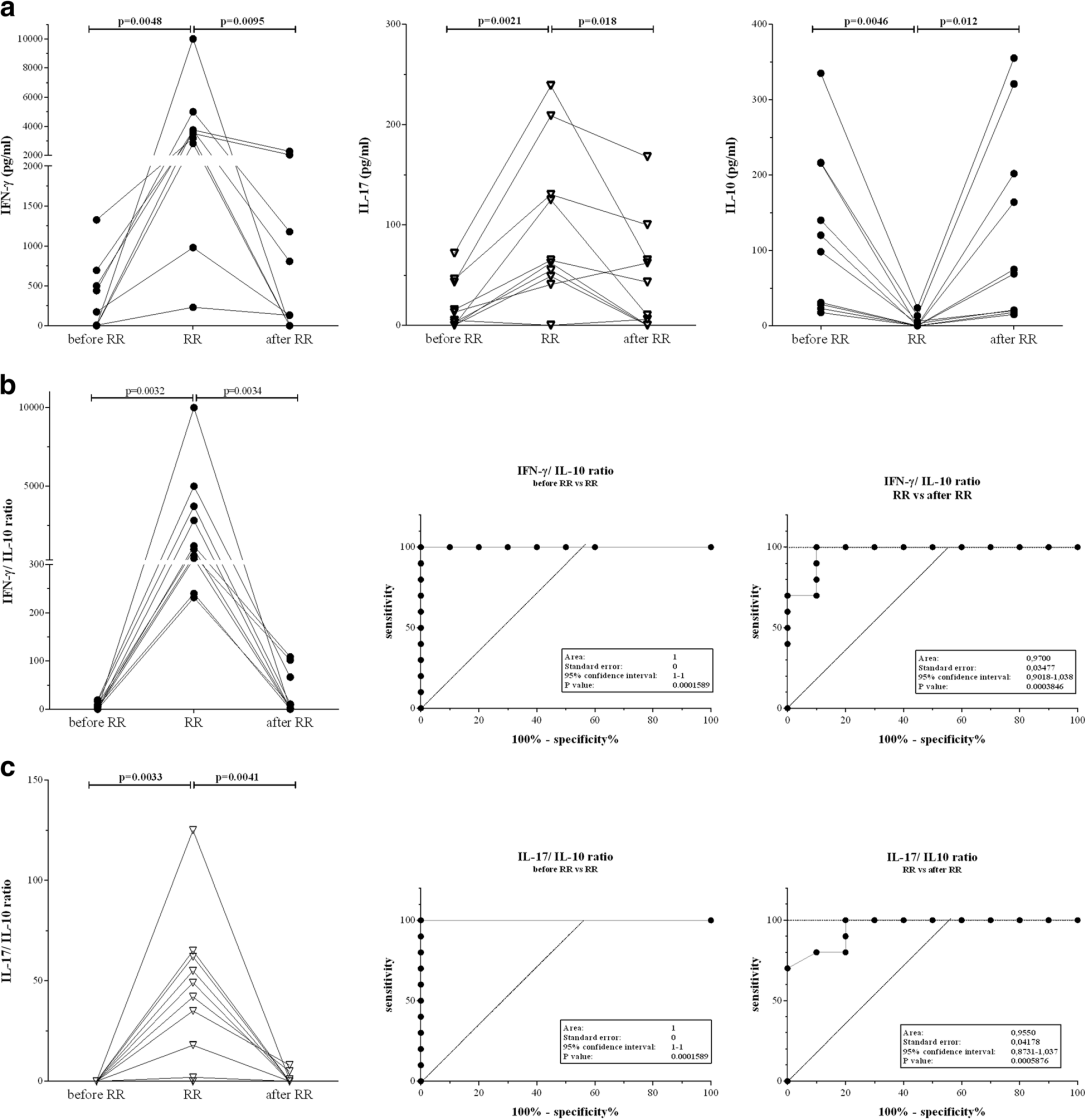
Longitudinal pattern of cytokine ratios for patients with reversal reaction (RR) IFN-γ, IL-17 and IL-10 production was induced by stimulation with *M. leprae* **a** for 10 patients who developed RR during this study (Bangladesh: n = 3; Brazil; n = 4; Nepal: n = 3) at leprosy diagnosis before MDT in the absence of any clinical signs of reactions and at least three months before reaction (*before RR*), at diagnosis of reaction before steroids (*RR*) or after MDT and RR, at least one month after end of steroids (*after RR*). IFN-γ/ IL-10 **b** and IL-17/ IL-10 **c** ratios and ROC (*receiver operating characteristics*) curves are shown. For calculations of ROC values, time points before RR versus at RR diagnosis (*B, C middle panels*) or at RR diagnosis versus after RR (*B, C right panel*) were considered

To identify biomarkers for early type 1 reactions, blood of newly diagnosed, untreated leprosy patients was obtained longitudinally in Bangladesh, Brazil, Ethiopia and Nepal (Table 1). The analysis included two samples of patients without reactions [1. before treatment (t = 0); 2. after treatment (t = end)] and three of patients who developed RR during the study [1. in the absence of clinical signs of reactions, at least 3 months before RR diagnosis (t = 0); 2. at RR diagnosis, before steroid-treatment (t = ×); 3. after RR and at least one month after ending steroid-treatment (t = end)]. Since patients were frequently diagnosed with RR at their first clinic-visit, it became clear that it was not always feasible to include these first samples. Initially, patients who developed RR within 3 months of recruitment were excluded to avoid measuring markers for RR already at t = 0. Similarly, patients showing clinical signs of reactions within 3 months after ending MDT and/ or steroid treatment were excluded to prevent measuring biomarkers of RR at t = end. For longitudinal analysis (Fig. 1) only patients entering the study without reactions were utilized. Due to the low frequency of untreated cases without RR at their first clinic visits who developed RR during this study, we also included patients with RR at their first clinic visits (as initial RR cases) consequently lacking the first time point (t = 0). Patients included in the analysis after database cleaning at each site are indicated in Table 1 and Figs. 3 and 4. For healthy individuals from these areas with identical socio-economic background, one sample was collected.

### Longitudinal *M. leprae-*induced cytokine/chemokine production during reaction development

First, we analysed *M. leprae-*induced cytokine production by blood cells of RR patients for whom valid samples were available at three time points (Bangladesh: *n* = 3; Brazil: *n* = 4; Nepal: *n* = 3). All patients produced significantly higher IFN-γ and IL-17 at RR diagnosis than before or after treatment (Fig. 1). Also, levels of IP-10, VEGF and IL-1β peaked at RR-onset (Additional file 2: Figure S2). In contrast, IL-10 was virtually not produced at RR diagnosis, compared to before diagnosis and after treatment. Cytokine responses to *M. leprae*-unique proteins, in particular ML2478 [23], corresponded well with responses to *M. leprae* (Additional file 2: Figure S1).

Since cytokines modulate each other’s effects, we considered ratios as markers for disease-status. Indeed, the differential cytokine production at RR onset was even more evident from the ratios of IFN-γ/IL-10 and IL-17/IL-10 (*p* = 0.0032; *p* = 0.0033; Fig. 1), whereas IFN-γ/IL-10 for patients who did not develop reactions remained similar before and after treatment (Additional file 2: Figure S3D) due to the simultaneous increase of both IFN-γ and IL-10 after MDT treatment in non-reactional patients (Figs. 3 and 4). The potential of cytokine ratios for discrimination between RR and its absence was also evident from the ROC (receiver operating characteristics) with AUC (areas under curve) ranging from 0,955–1. IP-10/IL-10 ratios showed a similar profile, with slightly less significance (AUC:0.79; Additional file 2: Figure S3B-C). Thus, cytokine ratios proved valuable, RR-associated markers as well as markers for reactional treatment efficacy.

### Longitudinal serological analysis during reaction development

**Fig. 2:**
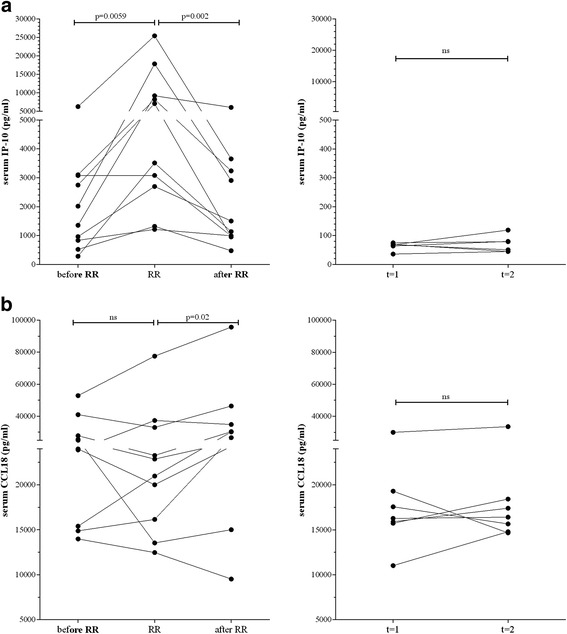
Longitudinal serum analysis of patients with reversal reaction (*RR*) Levels of IP-10 **a** and CCL18 **b** in unstimulated sera derived from 10 leprosy patients (*left panels*) developing RR (Bangladesh: n = 4; Brazil; n = 3; Ethiopia: n = 1; Nepal: n = 2) in the absence of any clinical signs of reactions and at least three months before reaction (*before RR*), at diagnosis of reaction before steroids (*RR*) or after MDT and RR, at least one month after end of steroids (*after RR*), or from healthy Dutch controls (*n* = 10) at two sequential time points with six months intervals (*right panels*). For calculations of the ROC values, time points at least three months before RR and at RR diagnosis before steroids were considered. IFN-β levels for controls were not detectable

For detection of *M. tuberculosis* infection [26] and to indicate *M. leprae* exposure [23, 27], IP-10 was reported a useful marker. Notably, IP-10 is produced in large quantities facilitating its use in field-friendly test-platforms such as lateral flow [28]. IP-10 analysis of longitudinal sera of reactional patients showed increased levels during RR (Fig. 2: *p* = 0.0059; Additional file 2: Figure S4: AUC:0,79) consistent with previous studies [6, 29]. Upon anti-reactional treatment, serum IP-10 decreased (*p* = 0.002; Fig. 2a). In contrast, longitudinal sera from patients without reactions or healthy donors, as control for RR-specificity, showed no significant difference in IP-10, clearly designating IP-10 as a serological marker for RR (Fig. 2).

The dynamics of CCL18 (chemokine (C-C motif) ligand 18) serum levels, elevated in lepromatous leprosy [30], were also investigated for patients experiencing reactions (Fig. 2b), showing a decreasing trend at RR, increasing after treatment for most patients. CCL18 in healthy controls were much lower than for borderline lepromatous patients in line with recent findings [30].

In view of the reduction of IL-10 during RR, these sera were also analysed for the presence of IL-10-inducing IFN-β [31]. Although no significant differences were detected at RR compared to before onset, IFN-β decreased significantly after treatment (*p* = 0.006; Additional file 2: Figure S6).

### Cross-sectional analysis of cytokine production

**Fig. 3:**
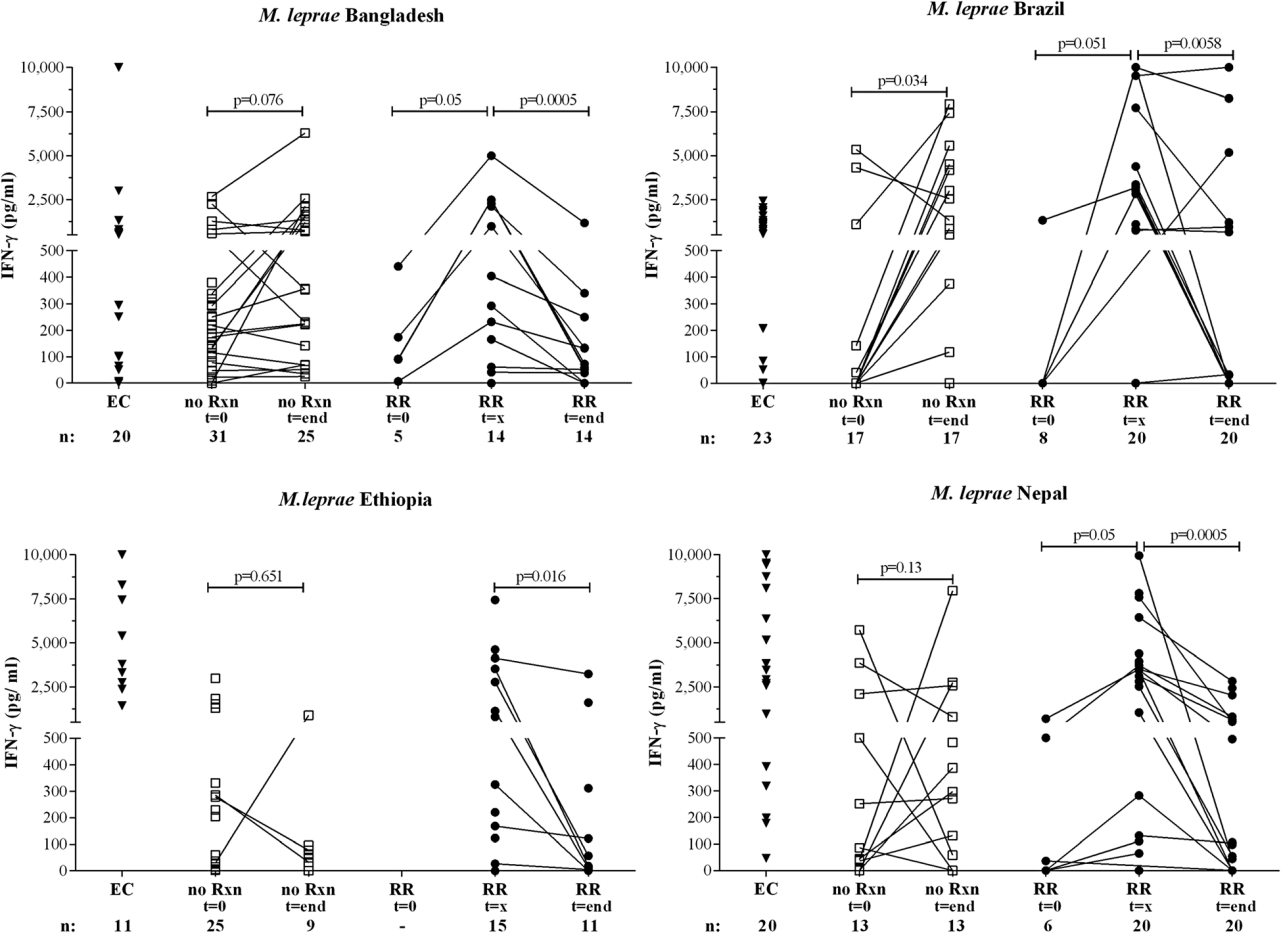
Longitudinal cross-sectional pattern of IFN-γ and IL-10 secretion. IFN-γ (Fig. 3) or IL-10 (Fig. 4) production (*corrected for background values*) in response to *M. leprae* sonicate (10 μg/ml) in 6 day cultures of peripheral mononuclear cells (*PBMC*) of endemic controls (EC; ▼), newly diagnosed leprosy patients without reactions (no Rxn;) before treatment (*t = 0*) and after treatment (*t = end*) and leprosy patients (•) in the absence of any clinical signs of reactions and at least 3 months before RR (*t = 0*), at RR diagnosis before steroids (*t = x*) or after MDT and RR (*t = end*), at least one month after end of steroids (*after RR*) in individuals from Bangladesh, Brazil, Ethiopia, and Nepal. All patients were assessed for the absence of reactions three months after t = end. Background values were typically < 50 pg/ ml. The number of individuals per group and the time point are indicated below the *x*-axis for each site

Cytokine profiles produced by blood cells cultured with *M. leprae* sonicate/-proteins [23] were analysed cross-sectionally as well (Fig. 3, Additional file 2: Figure S1, Additional file 2: Figure S2). In line with our longitudinal results, patients who developed RR produced significantly higher IFN-γ levels in response to *M. leprae* proteins at RR diagnosis than before onset of reaction or after reaction treatment regardless of their ethnic origin (blood at t = 0 from Ethiopian RR patients was not available). As found previously for leprosy-endemic areas, EC produced high IFN-γ levels to *M. leprae* [23, 27, 32, 33].

**Fig. 4:**
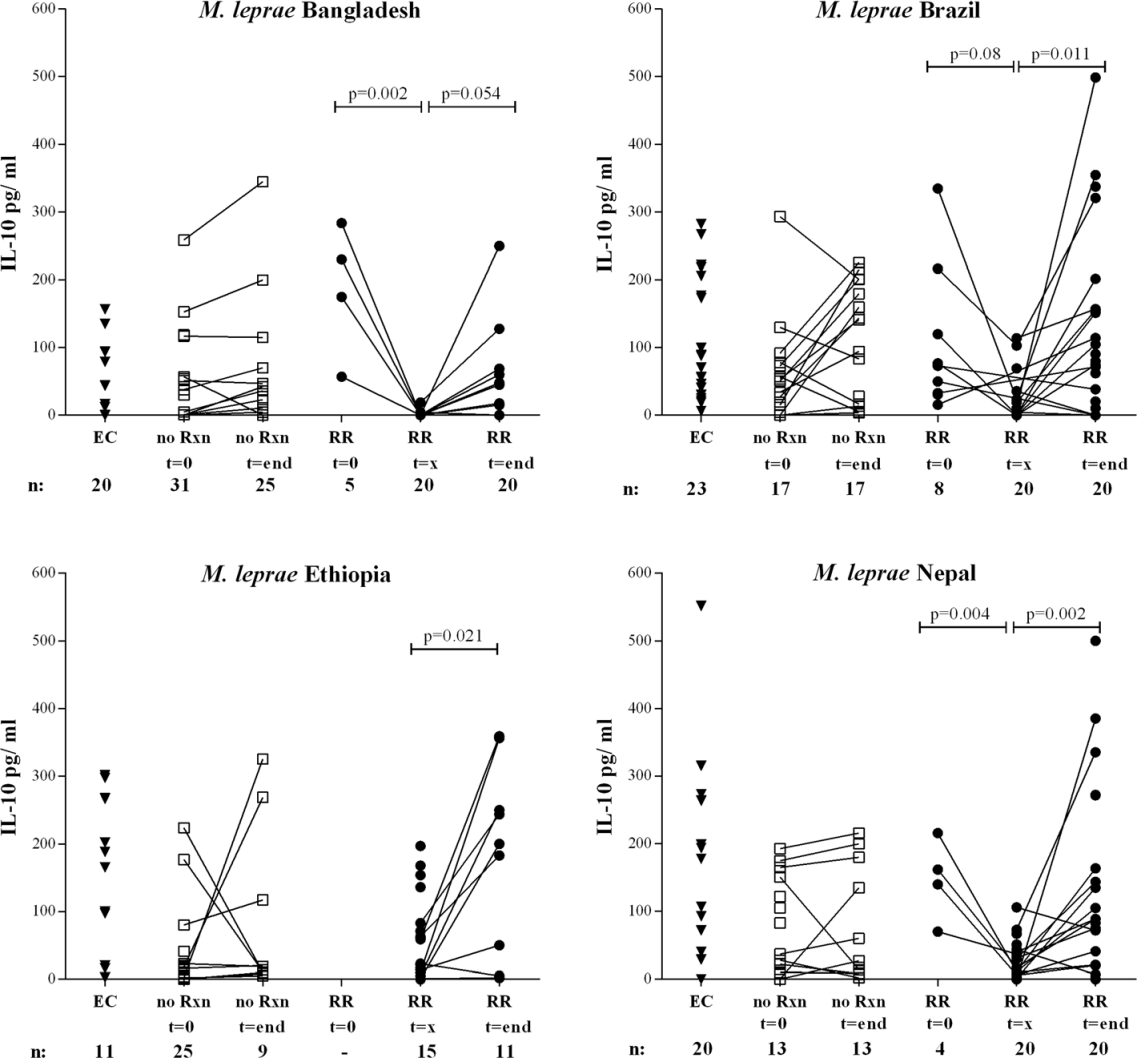
Longitudinal cross-sectional pattern of IFN-γ and IL-10 secretion. IFN-γ (Fig. 3) or IL-10 (Fig. 4) production (*corrected for background values*) in response to *M. leprae* sonicate (10 μg/ml) in 6 day cultures of peripheral mononuclear cells (*PBMC*) of endemic controls (EC; ▼), newly diagnosed leprosy patients without reactions (no Rxn;) before treatment (*t = 0*) and after treatment (*t = end*) and leprosy patients (•) in the absence of any clinical signs of reactions and at least 3 months before RR (*t = 0*), at RR diagnosis before steroids (*t = x*) or after MDT and RR (*t = end*), at least one month after end of steroids (*after RR*) in individuals from Bangladesh, Brazil, Ethiopia, and Nepal. All patients were assessed for the absence of reactions three months after t = end. Background values were typically < 50 pg/ ml. The number of individuals per group and the time point are indicated below the *x*-axis for each site

IL-10 levels in response to *M. leprae* were again in striking contrast to IFN-γ levels (Fig. 4). Virtually no responses were seen at RR diagnosis, compared to elevated IL-10 levels before diagnosis and after treatment. IP-10, IL-17, VEGF and to a lesser extent IL-1β levels followed those of IFN-γ, whereas G-CSF trended towards a decline at RR (Additional file 2: Figure S2). High levels of IL-5, IL-6, IL-8, MCP-1, MIP-1β, GM-CSF and TNF were observed for all groups but lacked a distinct longitudinal pattern, whereas induction of IL-2, IL-4, IL-7, IL-12p70 and IL-13 was low (data not shown).

### Biomarkers to monitor treatment efficacy

Besides biomarkers associated with reactions, biomarkers to monitor treatment efficacy provide practical tools as well. Thus, we analyzed the effect of treatment on immunemarkers: IFN-γ responses to *M. leprae* antigens of patients without reactions increased after treatment (Fig. 3), whereas IL-10 increased slightly, but not significantly with treatment (Fig. 4). Treatment-induced increasing trends were also observed for VEGF, IL-1β and IL-17A levels (Additional file 2: Figure S2) thereby contributing to the biomarker profile for RR. As observed for RR patients, IFN-γ levels also increased in patients without RR after MDT treatment. In contrast to reactional patients, however, IL-10 levels were higher after MDT which renders the drop in IFN-γ/IL-10 ratio (Fig. 1 and Additional file 2: Figure S3D) specifically associated with RR.

**Fig. 5:**
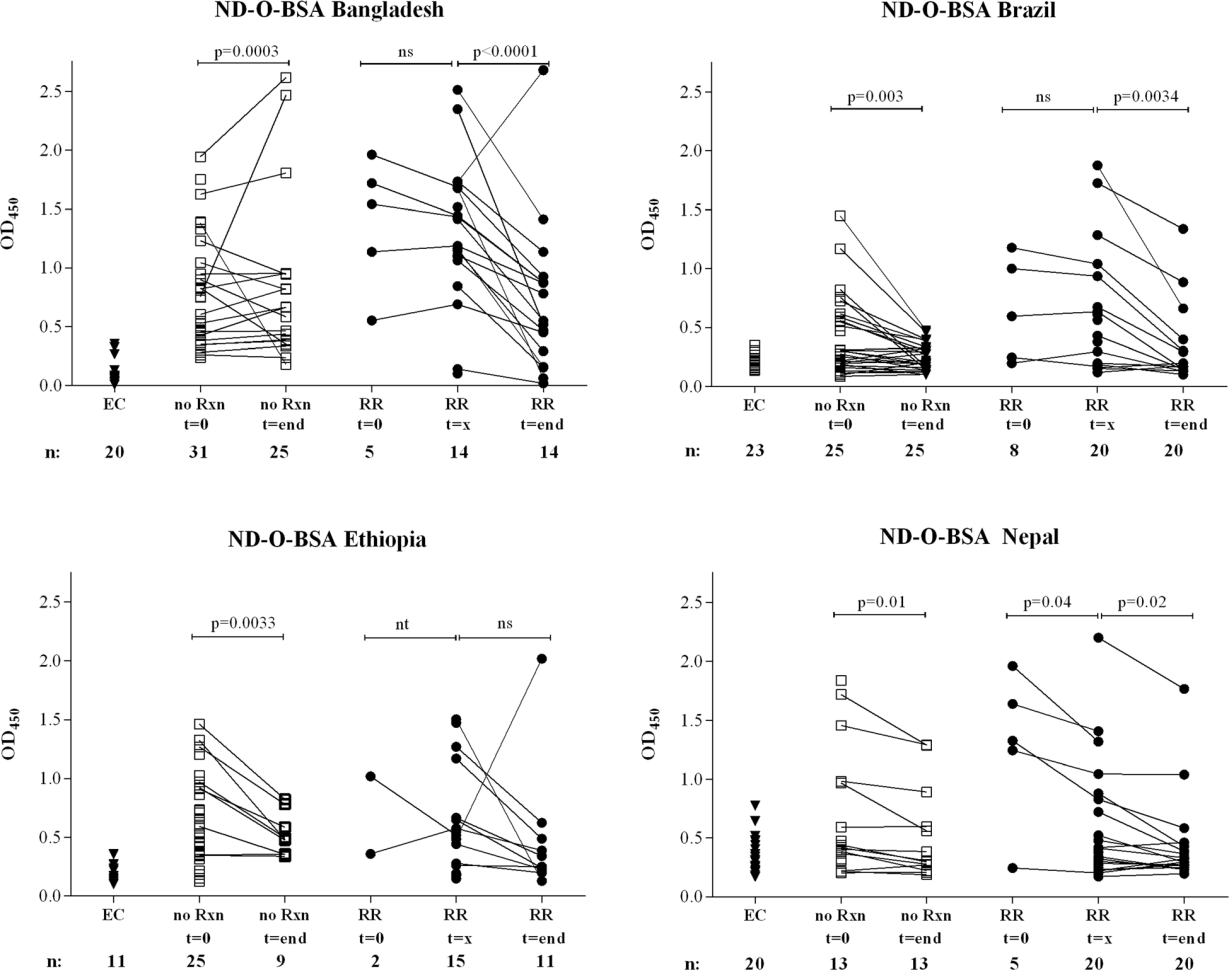
Humoral immunity to *M. leprae* antigens Antibodies against synthetic PGL-I (ND-O-BSA, a synthetic analog of the *M. leprae*-specific PGL-I) by ELISA. Sera were derived from Bangladesh, Brazil, Ethiopia, and Nepal and included endemic controls (EC; ▼), newly diagnosed leprosy patients without reactions (no Rxn;) before (*t = 0*) and after treatment (*t = end*) and leprosy patients (•) in the absence of any clinical signs of reactions and at least 3 months before RR (*t = 0*), at RR diagnosis before steroids (*t = x*) or after MDT and RR, at least one month after end of steroids (*t = end*). Optical density readings were performed using a 1:200 serum dilution. Median values for each group are indicated by horizontal lines. *P*-values < 0,05 indicate significant differences. The number of individuals per group and the time point are indicated below the *x*-axis for each site

Finally, cross-sectional screening of sera for the presence of antibodies to ND-O-BSA and ML2028 was performed (Fig. 5 and Additional file 2: Figure S5). Anti-PGL-I IgM levels, but not anti-ML2028 IgG levels were generally lowest in EC. In patients without RR, treatment significant decreased antibodies (*p* = 0.0003 – 0.01), confirming that these serological markers add to host profiles useful to estimate treatment [25]. However, *M. leprae-*specific antibody detection did not identify RR, but allowed treatment monitoring (*p* = 0.0001. – 0.02; the Ethiopian cohort did not reach significance), suggesting that humoral immunity could serve as auxiliary tool for monitoring reactional treatment in addition to serum IP-10 and IFN-β as well as cytokine ratio’s.

## Discussion

Biomarkers as reliable correlates of disease complications and response to therapy are essential tools for early diagnosis of disease states in chronic infections. Generally, the performance of one biomarker can be significantly enhanced by using instead a custom-made grouping of independent biomarkers, called a profile or signature. In the current situation of leprosy elimination, the availability of sensitive and specific biomarkers that aid early diagnosis of leprosy reactions as well as monitor therapy, would be a strategic advantage enabling health care workers to identify, treat and possibly prevent these episodes at early stages, thereby reducing nerve damage. Since the immunopathology of leprosy, particularly in reactional states, is linked to temporal changes in the immune response to *M. leprae*, leprosy represents a uniquely suitable model to study immune-biomarker changes in relation to clinical disease manifestations.

This is the first study in which cellular- and humoral immunity specific for *M. leprae* in leprosy patients within the three main continents reporting leprosy were monitored longitudinally during treatment. Although previous studies have analyzed circulating cytokines and chemokines [29] around the time of leprosy reactions. The addition of an *M. leprae* antigen-specific component, as utilized in this study, provides more specificity to this approach.

The data demonstrate translational importance since similar intra-individual trends were observed for development of RR in different endemic areas, allowing global application of these biomarkers in tests for early diagnosis of RR. In this respect, the importance of the combined effect of *M. leprae*-induced cytokine production (IFN-γ, IL-17, IP-10, IL-1β, VEGF), determined by their ratios versus IL-10, was highlighted, providing valuable tools for diagnosis of reactional states.

The biomarker profiles identified in this study for RR can be used in blood-based diagnostic tests [28] to detect (intra-individual) changes during these acute inflammatory periods but also provide an approach for other chronic diseases with acute inflammatory states such as tuberculosis [34] and buruli ulcer [35] (paradoxical reactions) and Crohn’s disease [36, 37], to help early diagnose such episodes thereby contributing to timely treatment and prevention of disease-specific tissue damage.

The acknowledged immunosuppressive role of IL-10 in lepromatous leprosy [38] as well as in *M. leprae* infected mice [39, 40] was also evident from its reduction at RR-onset [41]. Thus, during RR the breakdown of regulation, in favour of inflammation, seems to underlie the aetiology of reactional tissue damage, whereas balanced ratios of these immune responses, as present in nonreactional leprosy patients, are protective against RR [42]. This is in line with the associations of IL-10 genetic variants with development of leprosy and leprosy reactions [6, 43–46]. Suppression of IL-10 in a borderline tuberculoid-like murine model significantly augmented CD4/44^+^ and CD8/44^+^ longitudinal infiltrative responses specific to *M. leprae* antigens and permitted CD4^+^ T-cells to penetrate and fragment nerve [47], in line with our current field findings and supporting monitoring patient IL-10 levels in ratio to cytokines proven to escalate during RR as a potential early indicator of impending clinical RR.

As a second biomarker for RR in multiple ethnic backgrounds, increased serum IP-10 levels were identified, whereas CCL18, which is elevated in lepromatous leprosy [30], decreased at early RR in 6/10 patients who developed RR. Since CCL18 is secreted by dendritic cells upon recognition of *M. tuberculosis* [48] and has been implicated in differentiation of macrophages into an alternative phenotype [49] this suggests that decreased CCL18 levels lead to fewer alternatively activated macrophages and less T-cell regulation [6, 50]. These data therefore indicate that new biomarker discovery approaches for RR also contribute to our understanding of the RR-associated immunopathologic mechanisms, suggesting new opportunities for therapeutic interventions.

Since RRs are considered delayed hypersensitivity reactions caused by overreaction and/or dysregulation of host defence mechanisms, conscientious (personalized) treatment monitoring is vital similar to other diseases with acute inflammatory states such as psoriasis and Crohn’s disease which share specific susceptibility genes with leprosy [36, 51]. Our data showed that pro-inflammatory cytokine/IL-10 ratios, serum IP-10 can be used for monitoring treatment while not on steroids. Therefore, besides for early diagnosis of reactions, tests to monitor efficacy of treatment are useful as well, especially in the light of the reoccurrence of these episodes.

To allow access to diagnostic test at resource-poor field settings, we recently developed low-tech, robust lateral flow assays (LFAs) for (simultaneous) detection of inflammatory (IP-10) and regulatory (IL-10) immune responses together with anti-PGL-I IgM antibodies in short term whole blood assays [28, 52]. In the light of the currently identified immune markers for RR, field-friendly LFAs measuring these cytokines for leprosy patients on MDT at each clinic-visit may be helpful to early detect RR if used for intra-individual testing. Thus, to provide a rapid test, the diagnostic potential of the cytokine ratios defined here, need to be determined in future studies using whole blood assays as well.

## Conclusions

Type 1 or reversal reactions (RRs) are a major cause of leprosy-related nerve impairment and bear similarities with acute inflammation induced episodes in other (infectious) diseases. Since there is no laboratory test for the early diagnosis of these episodes, this multi-continental, longitudinal study on the occurrence of RRs in leprosy patients, showed for the first time that both *M. leprae*-specific cellular- as well as humoral host immune-profiles, correlating with early onset of these inflammatory episodes, can be identified. Biomarkers associated with diagnosis or efficiency of treatment of type 1 reactions were identified, based on intra-individual changes rather than single values. In particular, ratios of cytokines secreted by *M. leprae* stimulated blood cells as well as circulating cytokines in sera, contributed to these biomarker profiles. Thus, these profiles can be applied for the early diagnosis and to monitor reactional episodes and contribute to timely treatment and reduction/prevention of tissue damage.

## Additional files

Additional file 1:
**A systematic study on** ***M. leprae*** **antigen-induced host profiles for the identification of biomarkers for diagnosis of leprosy reactions.** (PDF 223 kb) Additional file 2: Figure S1. IFN-γ in response to *M.leprae*-unique protein ML2478 in 6 day cultures of PBMC (see Figure 3). Simultaneously, PBMC were cultured with proteins: ML0009, ML0121, ML0141, ML0188, ML1601, ML1976, ML1989, ML1990, ML2283, ML2478, ML2531, ML2532 and ML2567 (data not shown). **Figure S2.** IP-10 (**A**), TNF (**B**), IL-17 (**C**), VEGF (**D**) , IL1-β (**E**) and G-CSF (**F**) production in same cultures as described in Figure S1. **Figure S3.** IP-10 and IL-17 (**A**) after stimulation with M. leprae. IP-10/ IL-10 and IL-17/ IL-10 ratios are indicated (**B**, **C**). ROC curves were calculated for IP-10/ IL-10 and IL-17 /IL-10. Ratios for patients without reactions are shown as controls (**D**). **Figure S4.** IP-10 (**A**), IFN-β (**B**) and CCL18 (**C**) in sera. **Figure S5.** Antibodies against M.leprae protein ML2028 in sera determined by ELISA. Optical density readings were performed using a 1:200 dilution. Median values are indicated by horizontal lines. **Figure S6.** IFN-β in sera derived from patients developing RR in the absence of clinical signs of reactions and at least three months before reaction (**before RR**), at diagnosis of reaction before steroids (**RR**) or after MDT and RR, at least one month after end of steroids (**after RR**). For ROC values, timepoints at least three months before RR and at RR diagnosis before steroids were considered. IFN-β levels for controls were not detectable. (DOCX 260 kb)

## Abbreviations

AUC: Areas under curve
BB: Borderline-borderline
BL: Borderline-lepromatous
BT: Borderline-tuberculoid
EC: Endemic control
ENL: Erythema nodosum leprosum
G-CSF: Granulocyte colony stimulating-factor
GM-CSF: Granulocyte macrophage colony stimulating-factor
IFN: Interferon
IP-10: IFN-γ induced protein 10
IL: Interleukin
LL: Lepromatous leprosy
MCP-1: Monocyte chemoattractant protein 1
MDT: Multidrug therapy
MIG: Monokine induced by gamma interferon
MIP-1β: Macrophage inflammatory protein 1β
M. leprae: Mycobacterium leprae
PBMC: Peripheral blood mononuclear cells
PGL-I: phenolic glycolipd-I
ROC: Receiver operating characteristics
RR: Reversal reaction/ type 1 reaction
TB: Tuberculosis
TGF-β: Transforming growth factor β
TNFα: Tumor necrosis factor α
TT: Tuberculoid (TT) leprosy
VEGF: Vascular endothelial growth factor
WBA: Whole blood assay
WCS: Whole cell sonicate
