# Supplementary material for: Longitudinal immune profiles in type 1 leprosy reactions in Bangladesh, Brazil, Ethiopia and Nepal
Source: BMC Infect Dis. 2015 Oct 28;15:477. doi: 10.1186/s12879-015-1128-0 (PMC4625471; doi:10.1186/s12879-015-1128-0)
Supplement: Additional file 1: — A systematic study on M. leprae antigen-induced host profiles for the identification of biomarkers for diagnosis of leprosy reactions. (PDF 223 kb) [file 12879_2015_1128_MOESM1_ESM.pdf]

CASE REPORT FORM

CONFIDENTIAL

BIOMARKERS STUDY - NEPAL

A systematic study on *M.leprae* antigen-induced host profiles for the identification of biomarkers for diagnosis of leprosy reactions.

Medical Ethical Clearance number(s) NHRC: 751 and 445

Funding agency: Netherlands Leprosy Relief

Coordinating scientist Nepal:
Dr Deanna Hagge
Mycobacterial Research Laboratories
Anandaban Hospital
PO Box 151, Kathmandu, Nepal
Tel: +977-1-4290545; Fax: +977-1-4290538
E-mail: deannah@tlmnepal.org

Local study centres Nepal:
Study centre code 01
Anandaban Hospital
PO Box 151, Kathmandu, Nepal
Tel: +977-1-4290545; Fax: +977-1-4290538

Study centre code 02
LalGadh Leprosy Hospital
Janakpur District, Nepal

Coordinating scientist Netherlands:
Dr. Annemieke Geluk
LUMC, Dept. of Infectious Diseases
Albinusdreef 2, Building 1, K5, 23333 ZA Leiden
Tel: +31-71-5263844; Fax: +31-71-5216751
E-mail: a.geluk@lumc.nl

Case report form and database design:
Ron Schuring, Dr. Mirjam Bakker and Dr. Linda Oskam
KIT Biomedical Research
Meibergdreef 39, 1105 AZ Amsterdam, the Netherlands
Tel +31-20-5665446; Fax +31-20-6971841
E-mail: L.Oskam@kit.nl

Local data management:
Saraswoti Khadge or Kapil Neupane
Mycobacterial Research Laboratories
Anandaban Hospital
PO Box 151, Kathmandu, Nepal
Tel: +977-1-4290545; Fax: +977-1-4290538
E-mail: immuno@tlmnepal.org; kapiln@tlmnepal.org

CONTENTS OF FILE

General outline	1 page
1. Registration form	2 pages
2. Informed consent form	2 pages
3. Clinical data at intake	1 page
4. Blood and urine collection at intake	1 page
5. Reaction status at intake & follow-up	2 pages
6a. Reaction form Anandaban	1 page
6b. Reaction scoring sheet	2 pages
7. Blood and urine collection at start reaction	1 page
8. End of study form	1 page
9. Blood and urine collection at end of study	1 page
10. General remarks	1 page

Always fill in the registration form, even when the patient is going to be excluded!!!

GENERAL OUTLINE

Aim of the study

The aim of the BIOMARKERS – NEPAL study is to identify parameters that can predict the occurrence of leprosy-related reactions and nerve function impairment. We will study demographic, clinical, immunological and genetic parameters that may be involved in the development of leprosy reactions.

Study outline

Intake
New PB and MB leprosy patients (except TT patients without reactions) who are not on TB, or steroids treatment at intake will be included in the study. Leprosy patients who are already on MDT for more than 7 days, but do not have a reaction at intake will be excluded. Blood and urine will be collected for baseline data.

Follow-up
The patients will be followed for one year to see whether they develop reactions or new nerve function impairment.

Reaction
If the patient develops a reaction, data, blood and urine will be collected. With the blood and urine we can determine which components of the immunological profile of the patient have changed. At the end of the reaction we will also collect blood and urine to see whether the immunological profile has gone back to normal.

End of the study
Even if there are no reactions, blood and urine will be taken at the end of the study period (1 year) as part of the final assessment. Patients who remain in recurrent reaction for the whole year will be excluded. As some patients may return a little later than after 1 year, a period of up to 15 months is acceptable.

Collaboration

This project is a collaboration between Anandaban Leprosy Hospital in Nepal and Leiden University Medical Centre and KIT Biomedical Research in the Netherlands.

Role of the partners

Anandaban
The staff in Anandaban will include patients and controls, follow-up patients, collect clinical data and collect and process blood samples. They will also do the data entry for this part of the work. They will also train staff from Lal Gadh to do the same.

Leiden University Medical Centre
The staff in Leiden will test the samples on immunological and genetic parameters. They will also do the data entry for this part of the work. They will assist and train the partners in Nepal. They will also prepare the Standard Operating Procedures (SOPs) for the laboratory testing and coordinate the logistics of the sample collection, processing and shipment.

KIT Biomedical Research
The staff in Amsterdam will perform the serological testing of the samples. They will also do the data entry for this part of the work. They will coordinate the development of the case report form and the database and coordinate and facilitate the analysis of the data.

1. REGISTRATION FORM

Always fill in the registration form, even when the patient is going to be excluded!!! Please use capitals

Basic data

1. Patient name: _
2. Age in years: |_||_| *[yy]*
3. Sex: |_| *1 = male 2 = female*
4. Contact number/location: _
5. New patient or relapse: |_| *1 = new 2 = relapse (had complete course of MDT at least 2 years before intake)*

If relapse:

End date of last MDT	|_||_| / |_||_|	*[mm//yy]*
Had reactions during previous MDT course?	Yes / No	

Exclusion criteria *(fill in ALL exclusion criteria)*

Pure neural leprosy	Yes / No	*If yes, exclude*
TT leprosy	Yes / No	*If yes, exclude*
On treatment for TB	Yes / No	*If yes, exclude*
Receiving MDT for more than 7 days AND without reaction	Yes / No	*If yes, exclude*
Received steroid treatment in the last month	Yes / No	*If yes, exclude*
Planning to leave the area within 6 months	Yes / No	*If yes, exclude*
Under 18 or over 60 years old	Yes / No	*If yes, exclude*
Pregnant	Yes / No	*If yes, exclude*

1. REGISTRATION FORM

Inclusion in study

All exclusion criteria negative Yes / No *If no, exclude*

If "Yes" explain study and ask for written informed consent<br>*Fill in form 2*<br>*If "No" you can stop here*

Informed consent form signed Yes / No *If no, exclude*

If yes, add signed form 2 to file and proceed to forms 3 and 4<br>*Allocate patient study number*

6. Patient study number **BMS-**|_||_||_||_|

Patient Hospital file number |_||_||_||_||_||_||_|

2. INFORMED CONSENT FORM page 1 of 2

Patient study number BMS-|_||_||_||_|

Patient Hospital file number |_||_||_||_||_||_||_|

THE LEPROSY MISSION, NEPAL
ANANDABAN HOSPITAL
Bio-Marker Study

Patient consent form

(To be translated into appropriate local languages)

We wish to take a small amount of blood and urine from you, for use in a laboratory study. Approximately 20 ml of blood obtained using a needle and syringe and 10 ml clean catch mid-stream urine will be tested to see if your body is making an immune response to the leprosy bacteria.

This is an experimental test. Information obtained from this study may be useful in developing better ways of diagnosing early leprosy reactions and preventing it from consecutive deformities.

By your participation, you will be helping us to treat leprosy sufferers better. However if you do not wish to participate, your leprosy treatment will not be affected.

I have understood the above and give consent to have approximately 20 ml of blood and 10 ml of urine at one time for healthy endemic control and two time points for patients (three time if reaction occurred) for research purposes.

Signed ___________________________ Witness_________________________

Date ___________________________ Date _________________________

Staff who explained _______________________

Date _______________________

2. INFORMED CONSENT FORM

Patient study number BMS-|_||_||_||_|

Patient Hospital file number |_||_||_||_||_||_||_|

लेप्रोसी मिसन नेपाल, आनन्दबन अस्पताल

बायोमार्कर अध्ययन

सहभागीको अनुमती पत्र

सहभागीको नाम.. **अस्पताल नं**......................

प्रयोगशालामा गरिने जाँचको लागि तपाईंबाट अलिकती रगत र पिसाब दिन अनुरोध गर्दछौं। करिब २५ मिली जती रगत सिरीन्जको सहायताले लिनेछौं र यसको अलावा १० मिली सफा तरिकाले संकलन गरीएको पिसाब पनि लिइने छौं । यी रगत र पिशावका नमूनामा तपाइको शरिरले कुष्ठरोगको किटाणुको अंश संग कसरी प्रतिकृया गर्दछ भनेर हामो प्रयोगशालामा जाँच गरिने छ। यस अध्ययनले आउने रियाक्सनको समस्यालाई छिटै पत्तालगाई उपचार गर्न र अङ्गभङ्ग हुनबाट बचाउन मद्दत गर्न सक्ने छ।

यो एक प्रयोगात्मक जाँच हो। यस अध्ययनबाट प्राप्त जानकारीले भविष्यमा कुष्ठरोगको अझ राम्रो तरिकाले निदान गर्न सकिने प्रकृयाको विकास गर्नको लागि मद्दत पुऱ्याउन सक्नेछ

यस अध्ययनमा तपाईंको सहभागीताले तपाईं र तपाईं जस्ता अरु कुष्ठरोगबाट पिडित व्यक्तिहरुलाई अझ राम्रो तरिकाले उपचार गर्न हामीलाई मद्दत पुग्ने छ तैपनि तपाईं यस अध्ययनमा भाग लिन नचाहेमा पनि तपाईंले पाउँदै आएको उपचारमा कुनै असर पर्ने छैन ।

यस अध्ययनमा मेरो सहभागीता स्वऐच्छिक हो भन्ने कुरा मैले बुझेको छु २५ मि.लि रगत र १० मि ली पिशाव स्वस्थ व्यक्तिबाट एक पटक, बिरामीहरुबाट दुई पटक र यदि बिरामीको रियाक्सन आएमा तीन पटक अनुसन्धानको लागि दिन म राजी छु।

सहभागीको सहि ______________________ साक्षि ______________________

मिति ______________________ मिति ______________________

बुझाउने कर्मचारीको सहि ______________________

मिति ______________________

3. CLINICAL DATA AT INTAKE page 1 of 1

7. Date |_||_|/|_||_|/|_||_| *dd/mm/yy*

8. Ridley & Jopling classification (clinical) |_| *1=TT (exclude); 2=BT;3=BB;4=BL;5=LL;6= I*

9. Number of skin lesions |_||_| *If 1-15: give exact number*
If more than 15: code 99

10. Number of body parts involved |_| *There are 7 body parts: 2 arms, 2 legs, head, front trunk, back trunk*

11. Number of nerves involved |_||_| *Count the number of nerves involved*

12. Duration of signs and symptoms |_||_| *In months since reported first signs*

13. Microscopy on slit skin smears |_| *0=negative; 1=positive*

14. Average BI |_|.|_|

15. Ridley & Jopling classification (histological) |_| *1=TT;2=BT;3=BB;4=BL;5=LL;6= I*

16. Type of leprosy **treatment** |_| *1=PB; 2=MB > If PB and TT, exclude*

Ophthalmology

17. Leprosy-related ophthalmic impairment |_| *0=no; 1=yes*

18. Nerve function impairment |_|

0=no
1= nerve function reduction by ≥ 2 points in sensory and/or motor function tests
2= corneal anaesthesia
3= nerve tenderness
4=mixed mild symptoms of neuritis (nerve enlarged, sensory, and motor function scores of 1)
5= cutaneous neuritis
6= a mild skin reaction in a patch near or overlying a facial nerve.

Collect and process 20 ml of blood and 10 ml of urine according to SOP1-2 blood & urine processing. See form 4

4. BLOOD AND URINE COLLECTION AT INTAKE page 1 of 1

Collect and process 20 ml of blood and 10 ml of urine according to SOP1-2 blood & urine processing

Date of blood taking |_||_|/|_||_|/|_||_| *dd/mm/yy*

Heparine blood

Amount |_||_| *in ml*

Number of tubes |_|

Total yield

Plasma |_||_|.|_| *in ml*

Pax gene tube |_||_|.|_| *in ml*

PBMC |_||_|.|_| *in 10^6 cells*

Urine |_||_|.|_| *in ml*

Deviations from protocol

Processing within 3 hours of bleeding? |_| *0=yes; 1=no*

Processing at room temperature? |_| *0=yes; 1=no*

Storage at -20ºC? |_| *0=yes; 1=no*

For Pax gene tubes:

1 hr room temperature followed by freezing? |_| *0=yes; 1=no*

Label all tubes (serum, urine & Pax gene) and 96-well plate for supernatants with the following code make sure the handwriting is eligible and that marking sticks well, also if wet:

BMS-####-4

*patient study number*

4 *form number* *4=intake; 7=start reaction; 9=end of study*

5. REACTION STATUS AT INTAKE & FOLLOW UP page 1 of 2

Fill in 1 section (A,B,…) at intake and each time the patient is seen for treatment or complications

If reaction or new nerve function impairment: fill in form 6

After 1 year follow up proceed to form 8

A. Date: |_||_|/|_||_|/|_||_| *(dd/mm/yy)*

Reaction or new nerve function impairment Yes / No *If yes go to form 6*

Name doctor/health worker: ……………………………. Signature: …………………..

B. Date: |_||_|/|_||_|/|_||_| *(dd/mm/yy)*

Reaction or new nerve function impairment Yes / No *If yes go to form 6*

Name doctor/health worker: ……………………………. Signature: …………………..

C. Date: |_||_|/|_||_|/|_||_| *(dd/mm/yy)*

Reaction or new nerve function impairment Yes / No *If yes go to form 6*

Name doctor/health worker: ……………………………. Signature: …………………..

D. Date: |_||_|/|_||_|/|_||_| *(dd/mm/yy)*

Reaction or new nerve function impairment Yes / No *If yes go to form 6*

Name doctor/health worker: ……………………………. Signature: …………………..

E. Date: |_||_|/|_||_|/|_||_| *(dd/mm/yy)*

Reaction or new nerve function impairment Yes / No *If yes go to form 6*

Name doctor/health worker: ……………………………. Signature: …………………..

F. Date: |_||_|/|_||_|/|_||_| *(dd/mm/yy)*

Reaction or new nerve function impairment Yes / No *If yes go to form 6*

Name doctor/health worker: ……………………………. Signature: …………………..

G. Date: |_||_|/|_||_|/|_||_| *(dd/mm/yy)*

Reaction or new nerve function impairment Yes / No *If yes go to form 6*

Name doctor/health worker: ……………………………. Signature: …………………..

H. Date: |_||_|/|_||_|/|_||_| *(dd/mm/yy)*

Reaction or new nerve function impairment Yes / No *If yes go to form 6*

Name doctor/health worker: ……………………………. Signature: …………………..

5. REACTION STATUS AT INTAKE & FOLLOW UP page 2 of 2

Fill in 1 section (A,B,…) each time the patient is seen for treatment or complications

If reaction or new nerve function impairment: proceed to form 6

After 1 year follow up proceed to form 8

I. Date: |_||_|/|_||_|/|_||_| *(dd/mm/yy)*

Reaction or new nerve function impairment Yes / No *If yes go to form 6*

Name doctor/health worker: ………………………….. Signature: …………………

J. Date: |_||_|/|_||_|/|_||_| *(dd/mm/yy)*

Reaction or new nerve function impairment Yes / No *If yes go to form 6*

Name doctor/health worker: ………………………….. Signature: …………………

K. Date: |_||_|/|_||_|/|_||_| *(dd/mm/yy)*

Reaction or new nerve function impairment Yes / No *If yes go to form 6*

Name doctor/health worker: ………………………….. Signature: …………………

L. Date: |_||_|/|_||_|/|_||_| *(dd/mm/yy)*

Reaction or new nerve function impairment Yes / No *If yes go to form 6*

Name doctor/health worker: ………………………….. Signature: …………………

M. Date: |_||_|/|_||_|/|_||_| *(dd/mm/yy)*

Reaction or new nerve function impairment Yes / No *If yes go to form 6*

Name doctor/health worker: ………………………….. Signature: …………………

N. Date: |_||_|/|_||_|/|_||_| *(dd/mm/yy)*

Reaction or new nerve function impairment Yes / No *If yes go to form 6*

Name doctor/health worker: ………………………….. Signature: …………………

O. Date: |_||_|/|_||_|/|_||_| *(dd/mm/yy)*

Reaction or new nerve function impairment Yes / No *If yes go to form 6*

Name doctor/health worker: ………………………….. Signature: …………………

6A. REACTION FORM ANANDABAN

We will only include the first reaction that a patient develops

Start of reaction

Date of reaction	|_||_|/|_||_|/|_||_|	*(dd/mm/yy)*
Is this at intake?	Yes / No	
If no: time since start MDT	|_||_|	*in months*
Type of reaction	|_|	*1=Reversal reaction; 2=ENL; 3-neuritis*
Start dose prednisolon	|_||_|	*in milligrams*
Start date of prednisolon	|_||_|/|_||_|/|_||_|	*(dd/mm/yy)*
End date of prednisolon	|_||_|/|_||_|/|_||_|	*(dd/mm/yy)*

- *Fill in the reaction scoring sheet given in form 6B.*
- *Collect and process 20 ml of blood and 10 ml of urine according to SOP1-2 blood & urine processing. See form 7*

End of reaction

End of reaction treatment before end of follow up? |_| *0=yes; 1=no*

If yes:

Date last day reaction treatment	|_||_|/|_||_|/|_||_|	*(dd/mm/yy)*
Duration of reaction treatment	|_||_|	*in weeks*

If there are any observations relevant for the study and other than the ones on the form, please fill these in here

....

....

....

....

6B. REACTION SCORING SHEET

	Criteria	0	1	2	3	Score
A1	Degree of inflammation of skin lesions	**None**	**Erythema**	**Erythema and raised**	**Ulceration**	
A2	Number of raised and/or inflamed lesions	**0**	**1-5**	**6-10**	**>10**	
A3	Peripheral oedema due to reaction	**None**	**Minimal**	**Visible, but not affecting function**	**Oedema affecting function**	
**A SCORE**						

	**HANDS**	**Purple 2g Monofilament scores**				**Orange 10g Monofilament scores**			**Score**
	**Nerves**	**0**	**0.5**	**1**	**1.5**	**2**	**2.5**	**3**	
B1	RIGHT Trigeminal	**Felt**						**Not felt**	
B2	LEFT Trigeminal	**Felt**						**Not felt**	
B3	RIGHT ulnar	**All sites felt**	**1 site not felt**	**2 sites not felt**	**3 sites not felt**	**1 site not felt**	**2 sites not felt**	**3 sites not felt**	
B4	LEFT ulnar	**All sites felt**	**1 site not felt**	**2 sites not felt**	**3 sites not felt**	**1 site not felt**	**2 sites not felt**	**3 sites not felt**	
B5	RIGHT median	**All sites felt**	**1 site not felt**	**2 sites not felt**	**3 sites not felt**	**1 site not felt**	**2 sites not felt**	**3 sites not felt**	
B6	LEFT median	**All sites felt**	**1 site not felt**	**2 sites not felt**	**3 sites not felt**	**1 site not felt**	**2 sites not felt**	**3 sites not felt**	

	**FEET**	**Orange 10g Monofilament scores**				**Pink 300g Monofilament scores**			**Score**
	**Nerves**	**0**	**0.5**	**1**	**1.5**	**2**	**2.5**	**3**	
B7	RIGHT posterior tibial	**All sites felt**	**1 site not felt**	**2 sites not felt**	**3 sites not felt**	**1 site not felt**	**2 sites not felt**	**3 sites not felt**	
B8	LEFT posterior tibial	**All sites felt**	**1 site not felt**	**2 sites not felt**	**3 sites not felt**	**1 site not felt**	**2 sites not felt**	**3 sites not felt**	
**B SCORE**									

Date: |_||_| / |_||_| / |_||_| (mm/dd/yy) Name: ……………………………….. Signature: ……………..

6B. REACTION SCORING SHEET

	NERVE	0	1	2	3	Score
C1	RIGHT Facial	MRC =5	MRC=4	MRC=3	MRC<3	
C2	LEFT Facial	MRC =5	MRC=4	MRC=3	MRC<3	
C3	RIGHT Ulnar	MRC =5	MRC=4	MRC=3	MRC<3	
C4	LEFT Ulnar	MRC =5	MRC=4	MRC=3	MRC<3	
C5	RIGHT Median	MRC =5	MRC=4	MRC=3	MRC<3	
C6	LEFT Median	MRC =5	MRC=4	MRC=3	MRC<3	
C7	RIGHT Radial	MRC =5	MRC=4	MRC=3	MRC<3	
C8	LEFT Radial	MRC =5	MRC=4	MRC=3	MRC<3	
C9	RIGHT Lateral Popliteal	MRC =5	MRC=4	MRC=3	MRC<3	
C10	LEFT Lateral Popliteal	MRC =5	MRC=4	MRC=3	MRC<3	
**C SCORE**						

**Total score**	**Scores of A+B+C**	

This sheet is used to grade the severity of reactions. It has been developed by Walker et al. and published in PLoS Negl. Trop. Dis.,2008: 2(12): e351.

7. BLOOD AND URINE COLLECTION AT START REACTION

Collect and process 20 ml of blood and 10 ml of urine according to SOP1-2 blood & urine processing

Date of blood taking |_||_|/|_||_|/|_||_| *dd/mm/yy*

Heparine blood

Amount	|_||_|	*in ml*
Number of tubes	|_|	

Total yield

Plasma	|_||_|.|_|	*in ml*
Pax gene tube	|_||_|.|_|	*in ml*
PBMC	|_||_|.|_|	*in 10^6 cells*
Urine	|_||_|.|_|	*in ml*

Deviations from protocol

Processing within 3 hours of bleeding?	|_|	*0=yes; 1=no*
Processing at room temperature?	|_|	*0=yes; 1=no*
Storage at -20ºC?	|_|	*0=yes; 1=no*

For Pax gene tubes:

1 hr room temperature followed by freezing?	|_|	*0=yes; 1=no*

Label all tubes (serum, urine & PAXgene™) and 96-well plate for supernatants with the following code make sure the handwriting is eligible and that marking sticks well, also if wet:

BMS-####-7

*patient study number*

7 *form number* *4=intake; 7=start reaction; 9=end of study*

8. END OF STUDY FORM

The study ends 1 year (12 to 15 months) after the start of treatment

Date of assessment |_||_|/|_||_|/|_||_| *(dd/mm/yy)*

MDT Treatment completed |_| *Y=Yes; N=No*

How many monthly doses of MDT taken? |_||_| of |_||_| in |_||_| months

Did the patient develop reaction during the study period? |_| *0=No, 1=Yes*

Compare the ophthalmic and physiotherapy assessments of the beginning and the end of the study

Is the patient currently free of reactions? Yes / No

Is the patient currently under treatment for reaction? Yes / No

Were there new impairments during the study period? |_| *0=No, 1=Yes*

Are any of the previously existing impairments worse at the end of the study than at the beginning of the study? |_| *0=No, 1=Yes*

Other remarks

………

………

………

………

………

………

Collect and process 20 ml of blood and 10 ml of urine according to SOP1-2 blood & urine processing. Proceed to form 9

9. BLOOD AND URINE COLLECTION AT END OF STUDY

Collect and process 25 ml of blood and 10 ml of urine according to SOP1-2 blood & urine processing

Date of blood taking |_||_|/|_||_|/|_||_| *dd/mm/yy*

Heparine blood

Amount	|_||_|	*in ml*
Number of tubes	|_|	

Total yield

Plasma	|_||_|.|_|	*in ml*
Pax gene tube	|_||_|.|_|	*in ml*
PBMC	|_||_|.|_|	*in 10^6 cells*
Urine	|_||_|.|_|	*in ml*

Deviations from protocol

Processing within 3 hours of bleeding?	|_|	*0=yes; 1=no*
Processing at room temperature?	|_|	*0=yes; 1=no*
Storage at -20ºC?	|_|	*0=yes; 1=no*

For Pax gene tubes:

1 hr room temperature followed by freezing? |_| *0=yes; 1=no*

Label all tubes (serum, urine & Pax gene) and 96-well plate for supernatants with the following code make sure the handwriting is eligible and that marking sticks well, also if wet:

BMS-####-9

*patient study number*

9 *form number* *4=intake; 7=start reaction; 9=end of study*

10. GENERAL REMARKS

If there are any observations relevant for the study and other than the ones on the forms, please fill these in here

....
....
....
....
....
....
....
....
....
....
....
....
....
....
....
....
....
....
....
....
....
....
....
....
