# Supplementary material for: Longitudinal immune profiles in type 1 leprosy reactions in Bangladesh, Brazil, Ethiopia and Nepal
Source: BMC Infect Dis. 2015 Oct 28;15:477. doi: 10.1186/s12879-015-1128-0 (PMC4625471; doi:10.1186/s12879-015-1128-0)
Supplement: Additional file 2: Figure S1. — IFN-γ in response to M.leprae-unique protein ML2478 in 6 day cultures of PBMC (see Figure 3). Simultaneously, PBMC were cultured with proteins: ML0009, ML0121, ML0141, ML0188, ML1601, ML1976, ML1989, ML1990, ML2283, ML2478, ML2531, ML2532 and ML2567 (data not shown). Figure S2. IP-10 (A), TNF (B), IL-17 (C), VEGF (D) , IL1-β (E) and G-CSF (F) production in same cultures as described in Figure S1. Figure S3. IP-10 and IL-17 (A) after stimulation with M. leprae. IP-10/ IL-10 and IL-17/ IL-10 ratios are indicated (B, C). ROC curves were calculated for IP-10/ IL-10 and IL-17 /IL-10. Ratios for patients without reactions are shown as controls (D). Figure S4. IP-10 (A), IFN-β (B) and CCL18 (C) in sera. Figure S5. Antibodies against M.leprae protein ML2028 in sera determined by ELISA. Optical density readings were performed using a 1:200 dilution. Median values are indicated by horizontal lines. Figure S6. IFN-β in sera derived from patients developing RR in the absence of clinical signs of reactions and at least three months before reaction (before RR), at diagnosis of reaction before steroids (RR) or after MDT and RR, at least one month after end of steroids (after RR). For ROC values, timepoints at least three months before RR and at RR diagnosis before steroids were considered. IFN-β levels for controls were not detectable. (DOCX 260 kb) [file 12879_2015_1128_MOESM2_ESM.docx]

**Supplementary Information**

**Figure S1**: IFN-γ in response to *M.leprae*-unique protein ML2478 in 6 day cultures of PBMC (see Figure 3). Simultaneously, PBMC were cultured with proteins: ML0009, ML0121, ML0141, ML0188, ML1601, ML1976, ML1989, ML1990, ML2283, ML2478, ML2531, ML2532 and ML2567 (data not shown).

**Figure S2**: IP-10 (**A**), TNF (**B**), IL-17 (**C**), VEGF (**D**) , IL1-β (**E**) and G-CSF (**F**) production in same cultures as described in Figure S1.

**Figure S3**: IP-10 and IL-17 (**A**) after stimulation with *M. leprae*. IP-10/ IL-10 and IL-17/ IL-10 ratios are indicated (**B, C**). ROC curves were calculated for IP-10/ IL-10 and IL-17 /IL-10. Ratios for patients without reactions are shown as controls (**D**).

**Figure S4**: IP-10 (**A**), IFN-β (**B**) and CCL18 (**C**) in sera.

**Figure S5:** Antibodies against *M.leprae* protein ML2028 in sera determined by ELISA. Optical density readings were performed using a 1:200 dilution. Median values are indicated by horizontal lines.

**Figure S6:** IFN-β in sera derived from patients developing RR in the absence of clinical signs of reactions and at least three months before reaction (**before RR**), at diagnosis of reaction before steroids (**RR**) or after MDT and RR, at least one month after end of steroids (**after RR**). For ROC values, timepoints at least three months before RR and at RR diagnosis before steroids were considered. IFN-β levels for controls were not detectable.

**Supplementary Figures**

***Figure S1***

***Figure S2A (IP-10)***

***Figure S2B (TNF)***

***Figure S2C (IL-17)***

*** Figure S2D (VEGF)***

*** Figure S2E (IL1-β)***

***Figure S2F (G-CSF)***

******

***Figure S3***

******

***Figure S3D***

******

***Figure S4***

*** Figure S5***

******

***Figure S6***
